# The Relationship of Complex Post-traumatic Stress Disorder and Post-traumatic Stress Disorder in a Culturally Distinct, Conflict-Affected Population: A Study among West Papuan Refugees Displaced to Papua New Guinea

**DOI:** 10.3389/fpsyt.2017.00073

**Published:** 2017-05-31

**Authors:** Derrick Silove, Alvin Kuowei Tay, Moses Kareth, Susan Rees

**Affiliations:** ^1^Psychiatry Research and Teaching Unit, Academic Mental Health Unit, Liverpool Hospital, School of Psychiatry, University of New South Wales, Sydney, NSW, Australia

**Keywords:** complex post-traumatic stress disorder, post-traumatic stress disorder, complex grief, XI edition of the International Classification of Diseases, refugee

## Abstract

**Background:**

Controversy continues about the validity of the construct of complex post-traumatic stress disorder (C-PTSD). In particular, questions remain whether C-PTSD can be differentiated from post-traumatic stress disorder (PTSD) and, secondarily, other common mental disorders. The examination of these issues needs to be expanded to populations of diverse cultural backgrounds exposed to prolonged persecution. We undertake such an inquiry among a community sample of West Papuan refugees exposed to extensive persecution and trauma.

**Methods:**

We interviewed over 300 West Papuan refugees using the Refugee-Mental Health Assessment Package to record symptoms of PTSD, C-PTSD, major depressive disorder (MDD), and complex grief (CG). We used first- and second-order confirmatory factor analysis (CFA) to test aspects of the convergent and discriminant validity of C-PTSD.

**Results:**

The CFA analysis supported both a one-factor and two-factor model of PTSD and C-PTSD. Nested model comparison tests provide support for the parsimonious one-factor model solution. A second-order CFA model of PTSD and C-PTSD produced a poor fit. The modified three-factor multi-disorder solution combining a traumatic stress (TS) factor (amalgamating PTSD and C-PTSD), MDD, and CG yielded a good fit only after removing three CG domains (estrangement, yearning, and behavioral change), a model that produced large standardized residuals (>0.20).

**Conclusion:**

The most parsimonious model yielded a single TS factor combining symptom domains of C-PTSD and PTSD in this culturally distinct community exposed to extensive persecution and conflict-related trauma. There may be grounds for expanding the scope of psychological treatments for refugees to encompass this wider TS response. Our findings are consistent with theoretical frameworks focusing on the wider TS reaction of refugees exposed to human rights-related traumas of mass conflict, persecution, and displacement.

## Introduction

The notion that survivors of interpersonal trauma and abuse are at risk of developing a complex form of traumatic stress (TS) is long established in the literature (1–5). However, there has been an equally long legacy of controversy concerning the nosological status and composition of a proposed construct of complex post-traumatic stress disorder (C-PTSD) (6, 7). The intention to include a category of C-PTSD in the forthcoming XI edition of the International Classification of Diseases (ICD-11) therefore represents a turning point in the field, offering an opportunity to examine more critically key aspects of the validity of the construct.

By far the majority of past studies on C-PTSD in the field have been conducted among survivors of civilian trauma, particularly childhood sexual abuse (8). It is notable, however, that the first descriptions of a complex form of post-traumatic stress disorder (PTSD) were based on observations from treating survivors of persecution particularly WW-II concentration camps (9, 10). It is remarkable in that regard that in the modern era, relatively few inquiries into C-PTSD or its variants have involved populations exposed to mass conflict or torture, and the few that have done so have produced equivocal results in relation to the validity and utility of the diagnosis (11). It remains to be resolved therefore whether C-PTSD can be identified across cultures and, in particular, among societies exposed to persecution and displacement. A further issue is to define more clearly the relationship between PTSD and C-PTSD, noting that, by definition, the former has to be present to make a diagnosis of the latter. Within those definitional constraints, however, it is important to assess whether there is any differentiation between the two constellations, particularly among survivors of mass conflict, persecution, and displacement. The present study examines key aspects of the ICD-11 formulation of C-PTSD among a sample of West Papuan refugees displaced by prolonged persecution to Port Moresby, the capital of Papua New Guinea (PNG).

As indicated, the early literature contains rich descriptions of the adaptation and mental health symptoms of WW-II concentration camp survivors in the years following release from captivity (3, 12). From a modern perspective, the core symptoms identified spanned typical symptoms of PTSD, for example, nightmares, irritability, and sleep disturbances but also more general somatic, affective, and cognitive symptoms (fatigue, dysphoric moodiness, pervasive anxiety, memory, and concentration problems), features that would be distributed across a range of common mental disorders in modern classification systems; as well as persisting changes in behavior (loss of initiative, social withdrawal, and feelings of insufficiency or inadequacy) that reflect impairments in functioning (13). The general and wide-ranging nature of these latter symptoms present special obstacles to the endeavor of defining a more complex form of PTSD which is at least relatively independent of both PTSD and other common reactions to trauma (depression, prolonged grief).

The introduction of PTSD as an operationally defined category in DSM-III was a turning point in the nosology of the TS disorders but one which also accentuated polarizations in the field, particularly in intensifying calls for an additional if related category of C-PTSD. Advocates of C-PTSD argued that the symptom domains of PTSD [re-experiencing, avoidance (Avd) and numbing, and hyperarousal (Hyp)], although central to the core fear response, failed to encompass the wide range of reactions and adaptations exhibited by survivors of repeated and/or prolonged forms of interpersonal abuse (8). Based on a loose clinical consensus, the domains that were broadly considered to be part of C-PTSD included *inter alia*, dissociation, somatization, chronic/explosive anger, affective dysregulation (AD), poor impulse control and self-harming tendencies, compulsiveness, social withdrawal and isolation (3). This diverse array of characteristics was subsumed under the provisional umbrella diagnosis of disorder of extreme stress not otherwise specified (DESNOS) (14) proposed but ultimately excluded from DSM-IV after field trials failed to support the validity of the category (15).

By contrast, ICD-10 included the category of enduring personality change after catastrophic events (EPCACE) (16), the emphasis in that formulation being a persisting pattern of maladaptive attitudes and behaviors arising from exposure to abuses such as torture, persecution, and other chronic situations of abuse (17, 18). Although there were some commonalities, the differences between DESNOS and EPCACE perpetuated the divergence in formulations concerning the core elements of a putative C-PTSD construct, a key issue being whether the term referred to a constellation of symptoms (19, 20), or to deeper and enduring characterological changes occurring in reaction to prolonged exposure to gross forms of human rights abuse (18, 20).

The formulation of C-PTSD in ICD-11 represents a further attempt to derive a consensus position concerning the core elements of the construct (21). The organizing principle underlying this conceptualization of C-PTSD is that the response represents a disturbance in self-organization arising from exposure to chronic or recurrent forms of extreme interpersonal trauma and abuses. The three constituent domains of C-PTSD comprise AD, negative self-concept (NS), and disturbed relationships (15, 22). A fundamental assumption underlying the diagnosis is that C-PTSD is a variant of PTSD; PTSD therefore has to be present to consider making an added diagnosis of C-PTSD, thereby establishing a hierarchical relationship between the two constructs. From a methodological perspective, however, the mandated relationship between C-PTSD and PTSD creates major challenges in testing the nosological status of the former using standard approaches such as factor analysis, a limitation that is difficult to overcome.

Notwithstanding, researchers have commenced the task of examining key aspects of the ICD-11 category of C-PTSD. A latent profile analysis based on a study of survivors of interpersonal trauma in which symptoms of PTSD and C-PTSD were assessed yielded three classes: a C-PTSD class characterized by high scores on that constellation and PTSD, a PTSD only class in which survivors had minimal C-PTSD symptoms, and a low symptom class (23). Consistent with theory, chronic trauma was associated with C-PTSD, whereas exposure to a single trauma was related to PTSD. In two studies of survivors of early childhood sexual abuse, confirmatory factor analysis (CFA) supported a two-factor higher-order model of PTSD and C-PTSD, each construct comprising their underlying three symptom factors specified in ICD-11 (24, 25). Consistent with the ICD-11 system, PTSD was highly correlated with C-PTSD (*r* = 0.81). In a previous analysis of the present dataset based on a study among West Papuan refugees, we applied CFA to test the structure of PTSD and C-PTSD (26). The analysis supported the ICD-11 specified three-factor symptom structures of each category.

As indicated, the notion of a complex form of TS grew out of early observations of Holocaust survivors who had been exposed to gross abuses and torture in concentration camps during WW-II (9, 27). At that time, no distinction was made between what we now recognize as PTSD and the complex form, C-PTSD. An important question therefore is whether, among populations exposed to longstanding persecution and displacement, the two constellations (PTSD and C-PTSD) show both a degree of separation while retaining a pattern of close alignment or whether they combine to form a single structure. Guiding our inquiry is the finding of Cloitre and colleagues in a non-refugee population of trauma survivors that a single second-order factor can be identified comprising the dual constructs of ICD-11 domains of PTSD and C-PTSD (24, 25). An important question therefore is whether the same structure fits the pattern observed among refugees exposed to prolonged persecution; or, alternatively, whether the theoretical distinction between PTSD and C-PTSD is not supported in these populations but instead, represent a unitary construct comprising a combination of symptoms representing an expanded TS structure.

A related question is the extent to which C-PTSD can be discriminated from other common mental disorders. There is ample evidence of high levels of comorbidity of PTSD with disorders such as major depressive disorder (MDD) and complex grief (CG) in post-conflict populations worldwide, including in countries of the Former Yugoslavia (28), South-East Asia (29), and the Middle East (30). At face value, it seems plausible that some features of C-PTSD such as NS will overlap with or duplicate symptoms of self-blame and low self-worth typical of MDD; likewise the AD domain of C-PTSD may overlap with elements of complex grief (CG).

The present study examines more closely the relationship of PTSD and C-PTSD among the West Papuan refugee community, a population exposed to extensive persecution. During the decades’ long Indonesian occupation of West Papua, the indigenous community has experienced extensive traumas of mass conflict, including extra-judicial arrests, sexual violence, murder, and atrocities (31). Members of the independence movement are commonly apprehended and held in political prisons under harsh conditions of deprivation and abuse, including subjection to torture (32). Community members often are compelled to witness atrocities perpetrated against friends and family members (33). As a consequence of the persecution, successive waves of West Papuan refugees have crossed the border into PNG, settling in shanty towns surrounding Port Moresby (34), settings characterized by extreme poverty and lack of services. Our long-term interactions with the community confirmed that members had no prior familiarity with western concepts of trauma-related mental disorders such as PTSD, nor have they had contact with services providing interventions for trauma-related mental disorders.

In addition to our previous observations (26), we sought to examine (1) whether there was a particularly close association between C-PTSD and PTSD in this refugee population, reflected in a first-order factor structure in which the two constructs were highly correlated (testing for alternative two- and three-factor and higher-order models) and (2) whether there was evidence of specificity in the relationship of C-PTSD to PTSD indicated by lower levels of association between the former and MDD and complex grief (CG), respectively.

## Materials and Methods

### Sample

The community sample comprised 250 West Papuan refugees residing in 6 settlements in Port Moresby, PNG. In the absence of census data identifying members of this minority community within the larger population of PNG nationals, a targeted sampling approach was applied. In the first instance, based on all available sources of information (community leaders, government officials, international organizations, local university staff, and the United Nations High Commissioner for Refugees), we identified localities in which West Papuan refugees were concentrated. The six settlements are Hohola, Rainbow, Six Mile, Eight Mile, Nine Mile, and Tokarara/Waigani, localities with high density, make-shift (shanty) housing lacking basic facilities or services. Based on all available information, we estimated that 250 adults (90% of West Papuan refugees living in Port Moresby) resided in these settlements. In the second step, the study team mapped the location of all adult West Papuan refugees in the settlements by undertaking a comprehensive door-to-door survey coordinated by a West Papuan research assistant (Moses Kareth) from Australia who had long-term contact with the community. Twenty of those identified in the original list provided by informants had dispersed to other areas of Port Moresby or further afield, yielding a response rate (from the originally identified pool) of 92% (*n* = 230).

### Measures

Symptoms of C-PTSD, PTSD, complex grief (CG), and MDD were assessed using culturally adapted modules of the Refugee-Mental Health Assessment Package (R-MHAP) (35). Although the full R-MHAP also includes symptoms from the Diagnostic and Statistical Manual (editions IV and V) (14, 36), for clarity we restricted the analysis to ICD-11 symptoms (22) alone. We applied the entry criteria (exposure to traumas as specified) for PTSD and C-PTSD. The R-MHAP was developed to assess, *inter alia*, a wide range of common forms of mental disorders including PTSD, C-PTSD, major depression, anxiety-related disorders, prolonged grief, and explosive anger (35). In particular, at the initial stage of the development of the R-MHAP mental health assessment tool, we referred to both DSM (the IV and the proposed V editions) and ICD (the X and the proposed XI editions) classification systems as the blueprint for generating the core symptoms constituting each disorder category.

In relation to CG symptoms, respondents had to report a death or loss involving a family member and/or close friend in PNG, West Papua, or elsewhere in the past 12 months. Participants were asked to respond to the full list of symptoms for all four diagnostic categories under inquiry without applying skip rules. Items were scored “1” if present and “0” if absent, the sum of scores yielding a symptom count for each constellation in relevant analyses.

A process of consultation with community members and local psychiatrists was applied to test the cultural and contextual relevance of the mental health constructs under study as fully described previously (35). Although the indigenous psychiatrists we consulted were from PNG, they shared a common Melanesian background with the peoples of West Papua. All psychiatrists consulted reported utilizing the four mental health diagnoses in their practices with no major variation in the criteria specified in ICD-11. Focus groups included West Papuan refugees (*n* = 20) drawn from the full range of sociodemographic characteristics (ages, gender, education, and roles in the community). As expected, none were familiar with the formal nomenclature used to identify the index mental health constellations under study. However, symptoms and their significance were readily acknowledged as common and a source of distress within the community. Terms for symptoms of PTSD in the *lingua franca*, Bahasa Indonesian included “waspada” (hypervigilance), “menghindari” (Avd), “kehilangan minat” (loss of interest), “dijaga” (startle response), “sakit hati” (anger and resentment), and “tidak percaya” (loss of trust). Similarly, C-PTSD symptoms were recognized and named, for example, anger outbursts (“naik dadah”), self-blame (“meduduh diri”), detachment (“tersendiri”), and loss of interest (“kehilangan minat”). Participants were quick to acknowledge the presence of MDD symptoms in persons they knew, including low mood (“susah hati/sedih”), anhedonia or loss of interest (“kehilangan minat”), restlessness (“gelisah”), and fatigue (“kehilangan energy”). “Duka Cita” was the term that closely matched CG as an extreme reaction to interpersonal loss. In addition to endorsing typical CG symptoms, participants identified additional responses that were common among West Papuans, including confusion, a diminished sense of identity, and difficulties planning for the future. We have previously reported the identification of a six-factor model for the expanded pool of CG items (37), a structure that is similar to that found in a study of bereaved adults in the USA (22). The symptom subdomains identified for West Papuans included yearning/preoccupation, emotional distress, interpersonal dysfunction, shock and disbelief, negative appraisal, anger/bitterness, behavioral change, estrangement from others and impairment, and confusion and diminished sense of identity (37). For continuity and consistency, we used this item pool in the present analysis (unlike for the other three constellations where only ICD-11 criteria were applied).

Psychometric testing has attested to the validity and reliability of the R-MHAP as reported elsewhere (35). Internal reliability was high for all categories: PTSD, KR20 = 0.93; C-PTSD, KR20 = 0.88; MDD, KR20 = 0.94; and CG, KR20 = 0.94. A 6-month follow-up study of a subsample of 101 participants drawn from the baseline survey demonstrated a high degree of stability of the four symptom constellations over time (35).

### Procedure

Interviews were conducted by our field team of West Papuan refugees who received 3 weeks of intensive training under supervision of a bilingual clinical psychologist. Inter-rater reliability was assessed by the psychologist and a PNG medical practitioner psychiatric trainee who independently re-interviewed five study participants. A high rate of inter-rater agreement was achieved in assigning individual diagnoses between field workers and professional personnel (90% overall percentage agreement). Interviews were conducted in a private location or within the home of the participant, depending on their preference.

### Statistical Analysis

Pearson correlation coefficients were used to compare symptom counts of each of the four diagnostic constructs of C-PTSD, PTSD, MDD, and CG. Based on the approach of Hyland et al. (25), we undertook a series of confirmatory factor analyses (CFAs) as follows. To determine the factor structures of each symptom domain, we undertook first-order CFAs based on the summary scores derived from the constituent symptoms. For PTSD and C-PTSD, the analysis was informed by our prior examination of the data, which identified the ICD-11 structure comprising three symptom subdomains for each domain: for PTSD—intrusion (Int), Avd, and arousal; for C-PTSD—AD, NS, and interpersonal dysfunction (26).

To examine our hypotheses, we commenced testing one-factor (amalgamating PTSD and C-PTSD domains) into a broader TS factor and a two-factor model comprising domains of PTSD and C-PTSD. We also tested for a higher-order structure of PTSD and C-PTSD as reported by Cloitre and colleagues for a non-refugee population (25). This was followed by testing a three-factor multi-disorder structure of PTSD/C-PTSD, MDD, and CG. Chi-square tests of difference were conducted to examine statistical distinctions in model fit between the one-factor and two-factor models. In addition, we examined the CFA models for potential misspecifications and made iterative adjustments based on modification indices and normalized residuals (with standardized residuals of 0.20 or above signifying potential errors in model specification) (38).

Confirmatory factor analysis models were estimated using the robust mean- and variance-adjusted weighted least square method, an established statistical procedure recommended for analyzing dichotomous variables (39, 40), as applied extensively in past studies (41, 42).

Factor coefficients and correlations were calculated using a polychoric correlation matrix. In the analysis, we calculated standardized factor loadings and the covariance across factors. In general, a factor coefficient of 0.70 or above is considered to be a reliable indicator of a strongly loaded item; and a cross-factorial correlation of 0.90 or above indicates a high correlation between factors (43–45). We evaluated model fit using recommended goodness-of-fit and comparative indicators, including a non-significant chi-square test, the comparative fit index (CFI), Tucker–Lewis index (TLI), and root mean square error of approximation (RMSEA). A CFI and TLI greater than 0.95 and an RMSEA less than 0.06 indicates a good fit between the model and the data. A moderate fit is indicated by a CFI exceeding 0.90 and an RMSEA lower than 0.08. Analyses were performed using STATA version 13 (46) and Mplus version 7 (47).

## Results

### Sociodemographic Characteristics

The sample of West Papuan refugees comprised 230 adults (men 137, 59.5%; women 93, 40.4%), the mean age being 37 years (SD = 9.80). Approximately a half of the participants (107, 46.5%) were born in West Papua and the remainder (123, 52.4%) had spent all their lives residing in PNG. West Papuan born participants had lived in PNG for a mean of 27 years (SD = 10.28). Half of the participants resided in two settlements: Hohola (65, 28.2%) and Rainbow (47, 20.4%). Overall, based on symptom-based case assignments, 13% (*n* = 30) met criteria for ICD-10 PTSD, 6% (*n* = 14) for ICD-11 PTSD, and 3% (*n* = 8) for C-PTSD.

### Correlations and Symptom Counts of C-PTSD, PTSD, MDD, and CG

Table 1 examines correlations among the four mental health constructs based on the sum of their total symptom counts. The correlation was high between PTSD and C-PTSD (*r* = 0.62, *P* < 0.05); moderate for MDD symptoms with PTSD (*r* = 0.31, *P* < 0.05) and C-PTSD (*r* = 0.42, *P* < 0.05), respectively; and low between CG and PTSD (*r* = 0.27, *P* < 0.05) and CG and C-PTSD (*r* = 0.19, *P* < 0.05).

**Table 1:** **Correlation matrix of C-PTSD, MDD, PTSD, and CG according to symptom counts**.

	C-PTSD	PTSD	MDD	CG
C-PTSD	1.00			
PTSD	0.78*	1.00		
MDD	0.42*	0.40*	1.00	
CG	0.19*	0.30*	0.07	1.00

*PTSD, post-traumatic stress disorder; C-PTSD, complex post-traumatic stress disorder; MDD, major depressive disorder; CG, complex grief*.

*P < 0.05.

### Confirmatory Factor Analysis

#### One- and Two-Factor Models of PTSD and C-PTSD

**Figure 1:**
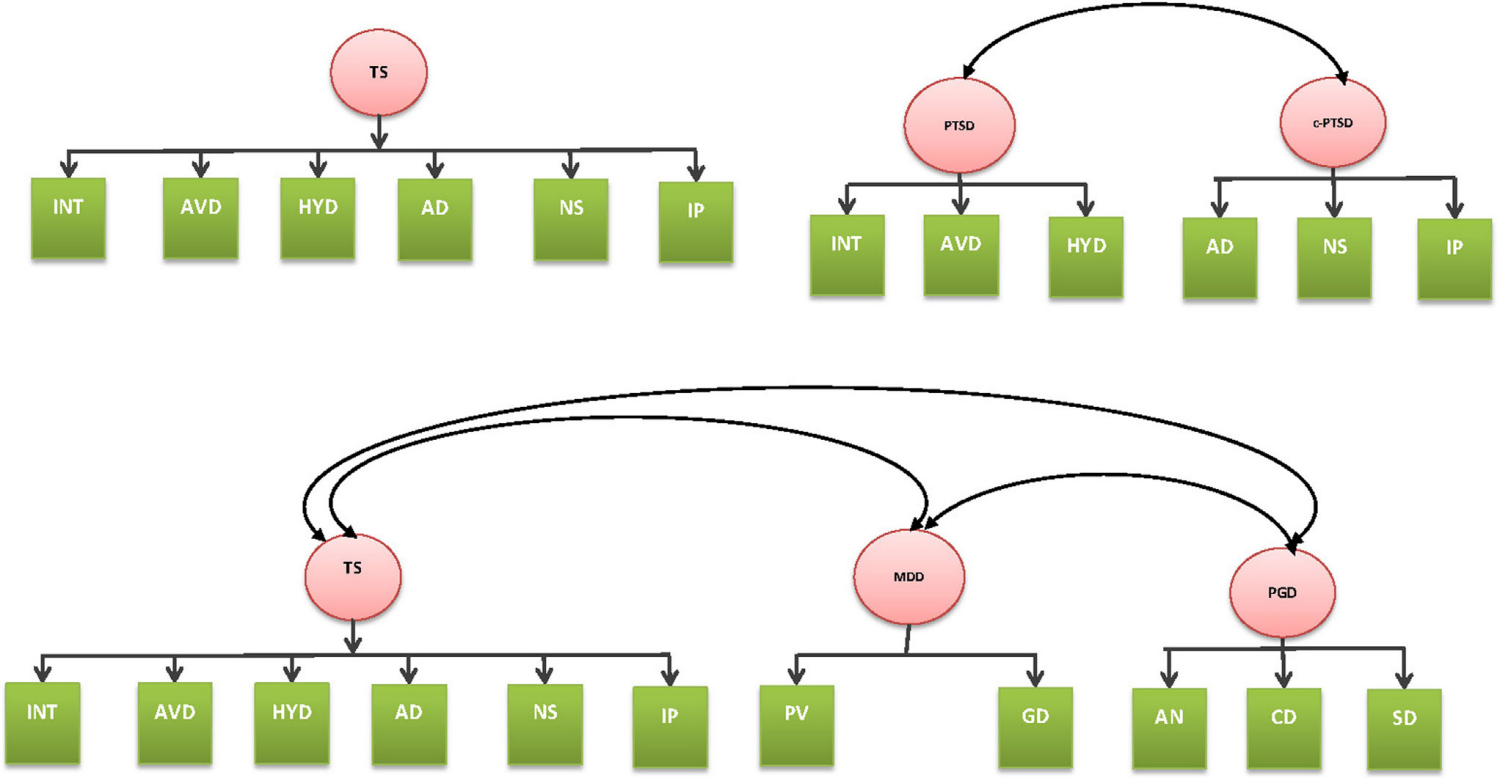
**One-factor and two-factor models of complex post-traumatic stress disorder (C-PTSD) and post-traumatic stress disorder (top)**. Three-factor model of C-PTSD, major depressive disorder, and complex grief (bottom).

Table 2 reports the goodness-of-fit statistics for the one- and two-factor models of PTSD and C-PTSD, respectively. Both the one-factor (χ^2^ = 7.33, *P* = 0.40, CFI = 1.00, TLI = 1.00, RMSEA = 0.01) and two-factor models of PTSD and C-PTSD (χ^2^ = 9.80, *P* = 0.28, CFI = 1.00, TLI = 1.00, RMSEA = 0.03) yielded a good fit. Standardized factor loadings for the CFA models are presented in Table 3. For both the one-factor and two-factor models (see Figure 1), the ICD-11 symptom subdomains consistently loaded onto the relevant latent construct (with a factor coefficient of 0.90 and above, although the Int domain had a slightly lower association with the PTSD construct in both one- and two-factor models). Specifically, the defined PTSD construct accounted for a considerable portion of the variance of the latent indicators of Ints, Avd, and Hyp; and the C-PTSD construct for AD, NS, and interpersonal difficulties.

**Table 2:** **Goodness-of-fit indicators for factorial models involving single and multiple disorder symptoms of PTSD, C-PTSD, MDD, and CG**.

Model	Description		Goodness-of-fit indices				
		χ^2^	df	*P*	CFI	TLI	RMSEA
1	One-factor traumatic stress model	7.33	7	0.40	1.00	1.00	0.01
2	Two-factor model of PTSD and C-PTSD	9.80	8	0.28	1.00	1.00	0.03
3	Three-factor model of PTSD/C-PTSD, MDD, and CG	339.08	72	0.001	0.88	0.85	0.13
4	Modified three-factor model of PTSD/C-PTSD, MDD, and CG^a^	53.90	39	0.06	0.99	0.99	0.04

*χ^2^, chi square; df, degree of freedom; CFI, comparative fit index; TLI, Tucker–Lewis index; RMSEA, root mean square error of approximation; PTSD, post-traumatic stress disorder; C-PTSD, complex post-traumatic stress disorder; MDD, major depressive disorder; CG, complex grief*.

*^a^We removed three domain indicators of yearning, estrangement, and behavioral change (with standardized residuals >0.20) from the CG construct*.

**Table 3:** **Standardized factor loadings for first-order confirmatory analysis of symptoms of PTSD and C-PTSD**.

	Est	SE	*R*
**One-factor confirmatory factor analysis (CFA) model**			
Traumatic stress (TS)			
Intrusion (Int)	0.68	0.09	0.46
Avoidance (Avd)	0.93	0.05	0.87
Hyperarousal (Hyp)	0.98	0.02	0.96
Affective dysregulation (AD)	0.93	0.03	0.87
Negative self-concept (NS)	0.92	0.03	0.85
Interpersonal problems (IP)	0.95	0.03	0.89
**Two-factor CFA model**			
PTSD			
Int	0.69	0.09	0.48
Avd	0.96	0.04	0.92
Hyp	0.98	0.03	0.93
C-PTSD			
AD	0.94	0.03	0.88
NS	0.92	0.03	0.85
IP	0.95	0.03	0.91
**Three-factor CFA model**			
TS			
Int	0.40	0.06	0.16
Avd	0.50	0.05	0.25
Hyp	0.92	0.02	0.84
AD	0.81	0.03	0.66
NS	0.83	0.03	0.69
IP	0.81	0.03	0.66
**MDD**			
Psycho-vegetative symptoms	0.89	0.04	0.78
General depressive symptoms	0.98	0.04	0.95
**CG**			
Anger and negative appraisal	0.89	0.04	0.79
Confusion and diminished identity	0.69	0.04	0.48
Shock and disbelief	0.72	0.04	0.52

*PTSD, post-traumatic stress disorder; C-PTSD, complex post-traumatic stress disorder; MDD, major depressive disorder; CG, complex grief*.

Convergent validity was supported by the high loadings of relevant symptoms on the two factors (PTSD and C-PTSD) comprising each construct (all ≥0.70). Nevertheless, the high cross-factorial correlation (0.95) between PTSD and C-PTSD suggests low discriminant validity between the two putative latent constructs and the chi-square test comparing the one-factor and two-factor models found no significant difference (χ^2^ diff = 2.47, *P* = 0.116). Based on the principle of parsimony, therefore, the findings support a one-factor general TS solution (comprising symptoms of ICD-11 defined PTSD and C-PTSD). It is also noted that the higher-order model tested by Cloitre et al. (25) failed to converge.

#### Multi-Category Three-Factor Model of C-PTSD, MDD, and CG

To test the second aim, based on the single factor structure identified in the preceding step, we undertook a three-factor multi-category model comprising the general TS construct (combined symptoms of ICD-11 defined PTSD and C-PTSD); two domains of MDD comprising psycho-vegetative symptoms (loss of appetite, insomnia, restlessness, fatigue, concentration difficulties) and non-specific depressive symptoms (low mood, anhedonia, self-blame, sense of worthlessness, and suicidal ideation); and the previously reported six-factor complex grief (CG) model. The analysis produced a poor fit [χ^2^(70) = 353.03, *P* < 0.001, CFI = 0.87, TLI = 0.84, RMSEA = 0.13]. Based on a detailed examination of the standardized residuals, we removed three CG symptom domains (yearning, estrangement, and behavioral change) because of their large residuals (>0.20). In addition, we included the covariance between the symptoms of Int and Avd, and between NS and Hyp. These modifications resulted in a significant improvement of the model fit [χ^2^(72) = 53.90, *P* = 0.06, CFI = 0.99, TLI = 0.99, RMSEA = 0.04]. In the modified three-factor model, the general TS factor (combined PTSD and C-PTSD symptoms) showed a moderate association with MDD (0.47, *P* < 0.01) and a relatively low association with CG (0.12, *P* < 0.01).

## Discussion

Our analysis provides broad support for a parsimonious single-factor model of general TS in this population, although a two-factor model (in which PTSD and C-PTSD were identifiable) produced an overall good fit. A three-factor model combining the latent constructs of general TS (PTSD and C-PTSD symptoms), MDD, and CG produced a good fit only when substantially modified. Importantly, in that model, the general TS factor showed only a moderate association with MDD and a low association with CG, although both were statistically significant.

A strength of the study is that it is one of only a few contemporary inquiries focusing on C-PTSD in a transcultural population exposed to extreme forms of persecution and human rights violations, thereby maintaining some continuity with earlier inquiries into the complex phenomenology exhibited by survivors of gross human rights violations dating back to WW-II (9, 10). Other strengths are the high response rate and the rigorous methodology we applied in developing and adapting our measures to the culture and context.

A limitation of the study is that it is restricted to a displaced West Papuan population residing in the specific context of the settlements in Port Moresby, PNG. Further studies among West Papuans living in other settings and across culturally diverse refugee groups worldwide will assist in testing the generalizability of our findings. Although we followed a systematic qualitative and quantitative approach in developing and adapting our measures to the culture and language (Bahasa Indonesian), there is always risk of transcultural error in applying measures, particularly in a community where literacy levels are low.

We selected CG and MDD as indicative categories to assess the convergent and discriminant validity of C-PTSD. It is possible that the inclusion of other categories, such as generalized anxiety disorder or panic disorder, known to be associated with PTSD, might yield different results. A general methodological challenge for the field, and one that is difficult to overcome, relates to the ICD-11 definition of C-PTSD. On the one hand, the category is defined hierarchically, requiring PTSD as a prerequisite of diagnosis; on the other hand, in order to establish the validity of C-PTSD it is essential to demonstrate its independence from other disorders. On undertaking our assessments, we followed the ICD-11 structure by inquiring into PTSD and C-PTSD in close sequence (35). There is a risk of an order effect bias in which participants respond in a uniform manner to items that follow in close sequence and that relate to similar issues. The consequence of such bias would be a spuriously high correlation between PTSD and C-PTSD based on procedural factors, an issue that would not be relevant for MDD and CG, constructs that are inquired into separately at different points in the interview. The difficulty in controlling for the hierarchical definition of PTSD and C-PTSD therefore continues to present a challenge to examining the nosological status of these constructs. Finally, we were obliged to generate specific symptoms to match the proposed ICD-11 definition of C-PTSD given that only a broad definition is specified according to the putative three subdomains (affective regulation difficulties; pervasive negative feelings about oneself; interpersonal dysfunction) (22).

Notwithstanding these caveats, our analysis found support for a single general TS factor although a two-factor model also demonstrated a good fit. It is noteworthy that we did not replicate the second-order structure for PTSD and C-PTSD reported by Cloitre and colleagues in studying a trauma-affected, non-refugee population in the USA (25). The difference in the findings between that study and our present data may be important in suggesting that refugee populations exposed to persecution and the traumas of human rights violations are distinctive in showing a general TS response in which ICD-11 specified PTSD and C-PTSD features are indissolubly represented. Such a finding would accord with the early descriptions of the wide-ranging TS response described among concentration camp survivors following WW-II, and possibly, with survivors of childhood abuse survivors, and former political prisoners (1, 3). Other studies have failed to support the distinction of PTSD and C-PTSD in relation to the number of traumas experienced (48). Together, therefore, the findings to date raise ongoing uncertainties as to whether a consistent distinction can be made between C-PTSD and PTSD. Nevertheless, our findings are consistent with a long-held view that survivors of multiple traumas related to gross abuses and human rights violations have a tendency to manifest a more general TS response comprising the characteristics of what are defined in ICD-11 as PTSD and C-PTSD.

On the face of it, the C-PTSD component of what we identified as a general TS factor appeared to share common features with MDD (for example, interpersonal difficulties) and CG (AD). Our three-factor model provided minimal support for this concern, however, by showing only a moderate association of the general TS factor with MDD and a low association with CG, falling within the bounds of comorbidity observed in general across disorders in psychiatry. We note, however, that several adjustments needed to be made to the model to achieve convergence, so the findings must be regarded as provisional. Further tests of these associations are warranted in other contexts and cultures.

There are several implications arising from to our findings. From a clinical perspective, it is possible that the assessment of symptoms among refugees has been excessively constrained to those of core PTSD. In that regard, it is notable that some studies undertaken among refugees in resettlement countries have documented a poor symptom response to conventional treatments for PTSD and depression (49, 50). The chronicity and complexity of the cases that receive treatment at specialized refugee-mental health services in these settings raise important questions whether the difficulties encountered in providing effective treatment relate in part to a failure to acknowledge the extent of the TS response which extends beyond core PTSD to encompass symptoms defined in ICD-11 as C-PTSD. It may be that refugees with this more general TS response which encompass difficulties in functioning and adaptation, warrant more extensive, multimodal approaches to treatment and rehabilitation which go beyond a focus on overcoming fear-related symptoms thought to underlie core PTSD (51, 52).

Complex post-traumatic stress disorder is formulated as a specific diagnostic category grounded in an individual-focused, conventional clinical system of classification. Concerns persist in the post-conflict field that a clinical diagnostic approach, while essential, needs to be embedded within a broader framework of understanding of a dynamically changing psychosocial context in which communities are exposed to a continuum of conflict, flight, and resettlement. The adaptation and development after persecution and trauma (ADAPT) model (52) proposes that both individual and communal responses to persecution and human rights violations occurring against a changing backdrop of disruptions of flight and resettlement undermine core psychosocial pillars of safety, bonds/networks, justice, roles and identities, and existential meaning (52). Within such a framework, psychopathology at the individual level represents an endpoint signaling a failure of adaptation arising from the complex interplay of personal vulnerability, past experiences, and ongoing conditions of adversity (53). Within such a framework, mental health symptoms are not regarded solely as stereotypic forms of independent pathology but as bearing understandable relationships with the types of traumas and psychosocial challenges that the individual and the collective confront. For example, PTSD and related anxiety symptoms reflect the human response to severe and repeated conditions of threat in settings where the person, and often the community, are not able to mobilize effective defensive strategies to mitigate the sense of danger (54). The corollary, however, is that the restoration of ecological conditions of safety can lead to a substantial decrease in PTSD symptoms, indicating the close reciprocal relationship between individual responses and population-wide indicators of security (55). Such a formulation cautions against the risk of reifying and individualizing trauma-related mental health categories such as PTSD and C-PTSD, the implication being that the problem is centered entirely within the individual rather than located within an interactional context involving the trauma survivor, the community, and the broader and ever-changing ecological context to which the displaced population is attempting to adapt.

## Conclusion

Our findings suggest that PTSD and C-PTSD symptoms as defined in ICD-11 may form aspects of one broad constellation of general TS in refugee communities exposed to prolonged mass persecution and displacement. Therefore, it is possible that the notion that PTSD and C-PTSD are related but somewhat distinct constructs (albeit hierarchically ordered) does not apply in populations exposed to multiple traumas associated with persecution. These findings need to be confirmed among other refugee groups. The next step is to integrate this broader conceptualization of the TS response into psychosocial formulations that fully represent the complexity of both the past and ongoing challenges experienced by communities exposed to prolonged persecution and forced displacement to adverse resettlement conditions.

## Ethics Statement

Ethical permission for the study was provided by the University of New South Wales Human Research Ethics Committee and the Medical Research Council of PNG Ethics Committee. Written consent and in some instances, witnessed oral consent were obtained from all participants prior to the interviews.

## Author Contributions

DS, AT, and SR contributed to the conception and design of the study. AT and MK conducted the study. AT performed the statistical analysis. DS and AT drafted the manuscript. All authors read and approved the final manuscript.

## Conflict of Interest Statement

The authors declare that the research was conducted in the absence of any commercial or financial relationships that could be construed as a potential conflict of interest.
